# Phenotypes of pain behavior in phospholipase C-related but catalytically inactive protein type 1 knockout mice

**DOI:** 10.1186/1744-8069-7-79

**Published:** 2011-10-18

**Authors:** Keisuke Migita, Masahiko Tomiyama, Junko Yamada, Masashi Fukuzawa, Takashi Kanematsu, Masato Hirata, Shinya Ueno

**Affiliations:** 1 Department of Neurophysiology, Hirosaki University Graduate School of Medicine, 5 Zaifu-cho, Hirosaki, Aomori 036-8562, Japan; 2 Department of Biofunctional Science, Faculty of Agriculture and Life Science, Hirosaki University, Aomori 036-8561, Japan; 3 Department of Dental Pharmacology, Graduate School of Biomedical Sciences, Hiroshima University, Hiroshima 734-8553, Japan; 4 Laboratory of Molecular and Cellular Biochemistry, Faculty of Dental Science, Kyushu University, Fukuoka 812-8582, Japan

## Abstract

Phospholipase C-related inactive protein (PRIP) plays important roles in trafficking to the plasma membrane of GABA_A _receptor, which is involved in the dominant inhibitory neurotransmission in the spinal cord and plays an important role in nociceptive transmission. However, the role of PRIP in pain sensation remains unknown. In this study, we investigated the phenotypes of pain behaviors in PRIP type 1 knockout (*PRIP-1 *^*-/- *^) mice. The mutant mice showed hyperalgesic responses in the second phase of the formalin test and the von Frey test as compared with those in wild-type mice. *In situ *hybridization studies of GABA_A _receptors revealed significantly decreased expression of γ2 subunit mRNA in the dorsal and ventral horns of the spinal cord in *PRIP-1 *^*-/- *^mice, but no difference in α1 subunit mRNA expression. β2 subunit mRNA expression was significantly higher in *PRIP-1 *^*-/- *^mice than in wild-type mice in all areas of the spinal cord. On the other hand, the slow decay time constant for the spontaneous inhibitory current was significantly increased by treatment with diazepam in wild-type mice, but not in *PRIP-1 *^*-/- *^mice. These results suggest that *PRIP-1 *^*-/- *^mice exhibit the changes of the function and subunits expression of GABA_A _receptor in the spinal cord, which may be responsible for abnormal pain sensation in these mice.

## Findings

Neuropathic pain is a critical disease that is induced by cancer, diabetes, infection or nerve damage [1]. Many studies have suggested that neuropathic pain occurs as a result of functional and/or anatomical changes in the primary sensory ganglia and the dorsal horn of the spinal cord after peripheral nerve injury [2-5]. One of these changes is the activation of microglia in the spinal dorsal horn. These microglia enhance the expression of neurotransmitter receptors and intracellular signaling kinases [6-8]. Changes in the expression of neurotransmitter (e.g., glutamate, ATP and GABA) receptors also occur in the dorsal root ganglion neurons or the spinal neurons [9-12]. GABA is the predominant inhibitory neurotransmitter in the spinal cord and in nociceptive transmission [13-16]. Accordingly, inhibition of GABAA receptors results in hypersensitivity of dorsal horn neurons and allodynia [17-20]. Therefore, reduced GABAergic input to the dorsal horn of the spinal cord is considered to be an important factor in the induction of allodynia.

Phospholipase C (PLC)-related inactive protein (PRIP) was first identified as a novel inositol 1,4,5-trisphospate [Ins(1,4,5)P3] binding protein, which is homologous to PLC-δ1, but is catalytically inactive [21]. The PIRP family consists of at least two types of proteins, PRIP-1 and PRIP-2. PRIP-1 is predominantly expressed in the brain, while PRIP-2 is ubiquitously expressed [22,23]. PRIP-1 has a number of binding partners that include the catalytic subunit of protein phosphatase 1α and GABAA receptor-associated protein [24]. This prompted us to examine the possible involvement of PRIP in GABA_A _receptor function. One role of PRIP-1 in regulating GABAA receptor functions was suggested based on the changes in GABAA receptor pharmacology and behavior in PRIP-1 knockout (*PRIP-1 *^*-/- *^) mice [24,25]. It is possible that *PRIP-1 *^*-/- *^mice exhibit abnormal pain sensation because of altered GABAA receptor functions in the central nervous system (CNS). Therefore, in this study, we examined the phenotypes of pain behavior and GABAA receptor subunit mRNA expression in *PRIP-1 *^*-/- *^mice.

**Figure 1:**
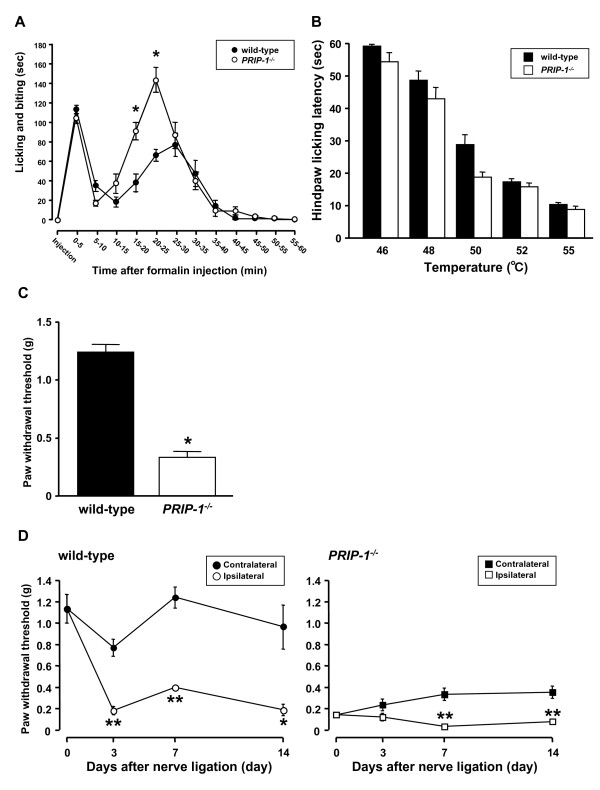
**Chemical, thermal and mechanical-induced pain and PSNL-induced tactile allodynia in *PRIP-1 *^*-/- *^ mice and wild-type mice**. (A) Duration of licking and biting responses for each 5-min interval after intraplantar injection of formalin in *PRIP-1 *^*-/- *^mice (n = 14) and wild-type mice (n = 10). Each point represents the mean and S.E.M. (*P < 0.05 vs. wild-type). (B) Hindpaw licking latency was measured at five temperatures (46, 48, 50, 52 and 55°C). Each value represents the mean and S.E.M. (n = 6-10). (C) Paw withdrawal threshold in response to tactile stimulation with von Frey filaments in *PRIP-1 *^*-/- *^mice (n = 6) and wild-type mice (n = 6). Each value represents the mean and S.E.M. (*P < 0.05 vs. contralateral side). (D) Paw withdrawal threshold in response to tactile stimulation with von Frey filaments in *PRIP-1 *^*-/- *^mice (n = 5-7) and wild-type mice (n = 8-14) before (0) and 3, 7 and 14 days after PSNL. Each point represents the mean and S.E.M. (*P <*0.05*, **P <*0.01 *vs. the contralateral side, n = 6-10).

To determine how PRIP-1 affects acute nociceptive hypersensitivity, we first performed the plantar formalin test in *PRIP-1 *^*-/- *^and wild-type mice (Figure 1A). Intraplantar injection of formalin evoked biphasic licking and biting behaviors. The first and second phases lasted 5 min and 60 min, respectively, after formalin injection. *PRIP-1 *^*-/- *^mice failed to show changes in the first phase, but the second phase response was significantly greater and the peak of the second phase occurred earlier in *PRIP-1 *^*-/- *^mice than in wild-type mice.

The hot plate test was performed at five different temperatures (46, 48, 50, 52 and 55°C) (Figure 1B). The latency to hindpaw licking was temperature-dependent and no difference was observed at 46, 48, 52 or 55°C between *PRIP-1 *^*-/- *^and wild-type mice. On the other hand, the mutant mice tended to be hypersensitive at 50°C, although this was not statistically significant.

To determine whether PRIP-1 plays a role in the behavioral responses to mechanical stimuli, the von Frey test was performed in *PRIP-1 *^*-/- *^mice and in wild-type mice (Figure 1C). Mechanical paw withdrawal thresholds were significantly lower in *PRIP-1 *^*-/- *^mice than in wild-type mice. Next, to investigate the influence of PRIP-1 on tactile allodynia during neuropathic pain, we performed partial sciatic nerve ligation (PSNL) in *PRIP-1 *^*-/- *^mice and in wild-type mice (Figure 1D). Wild-type mice showed a significant decrease in ipsilateral paw withdrawal threshold after PSNL, which started at least 3 days after PSNL and lasted for more than 10 days. Although *PRIP-1 *^*-/- *^mice showed greater hypersensitivity than wild-type mice, the *PRIP-1 *^*-/- *^mice also showed a strong decrease in ipsilateral paw withdrawal threshold after PSNL. This effect started at least 7 days after PSNL and lasted for more than 7 days.

**Figure 2:**
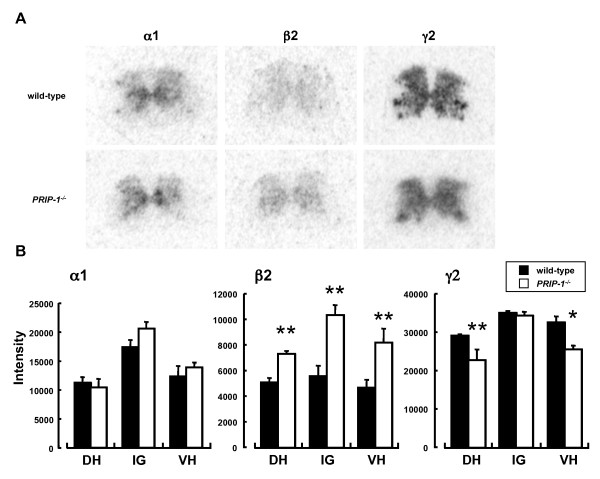
**mRNA expression of the GABA _A _receptor α1, β2 and γ2 subunits in the spinal cord of *PRIP-1 *^*-/- *^ mice and wild-type mice**. (A) Representative film autoradiograms showing the mRNA expression of GABA_A _receptor α1, β2 and γ2 subunits. (B) Semiquantitative analysis of GABA_A _receptor α1, β2, and γ2 subunit mRNA expression in the dorsal horn (DH), intermediate gray matter (IG) and ventral horn (VH) of the spinal cord in *PRIP-1 *^*-/- *^mice (black) and wild-type mice (white). Each value represents the mean and S.E.M. (*P < 0.05 , **P < 0.01 vs. wild-type, n = 3).

Previous studies showed that transport of the GABA_A _receptor γ subunit was altered in *PRIP-1 *^*-/- *^mice, thus reducing the sensitivity to benzodiazepine [25,26]. The majority of GABA_A _receptors in the CNS are believed to consist of α1, β2, and γ2 subunits [27]. Therefore, we investigated the mRNA expression patterns of these GABA_A _receptor subunits in the spinal cord of *PRIP-1 *^*-/- *^mice and in wild-type mice (Figure 2). α1, β2 and γ2 subunit mRNA expression was observed in the dorsal horn, intermediate gray matter and ventral horn of the spinal cord in these mice. There were no differences in α1 subunit mRNA expression between *PRIP-1 *^*-/- *^mice and wild-type mice. On the other hand, β2 subunit mRNA expression was significantly greater in *PRIP-1 *^*-/- *^mice than in wild-type mice throughout the spinal cord. In contrast, mRNA expression of the γ2 subunit was significantly decreased in the dorsal and ventral horns of the spinal cord in *PRIP-1 *^*-/- *^mice compared with wild-type mice.

**Figure 3:**
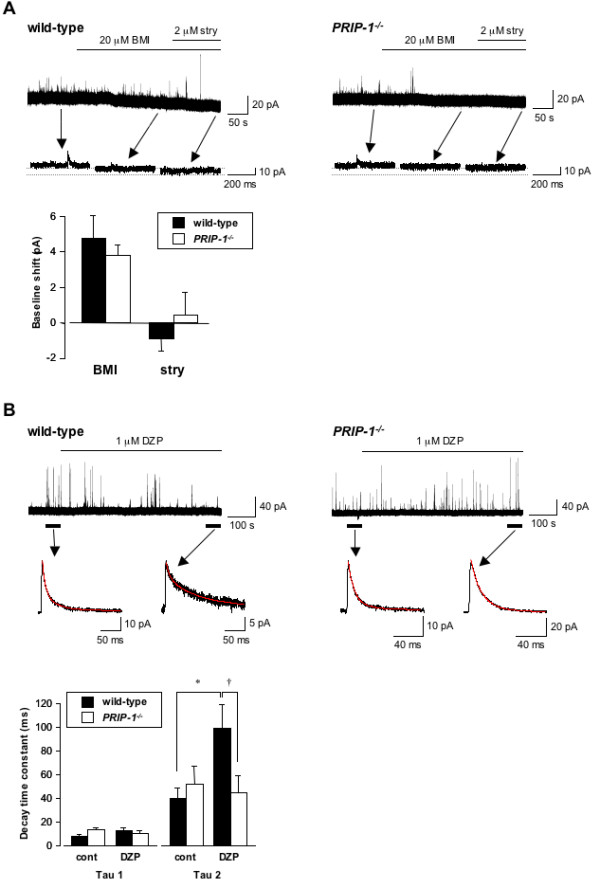
**Inhibitory synaptic and extrasynaptic currents in SG neurons of *PRIP-1 *^***-/- ***^ mice and wild-type mice**. (A) Representative traces and expanded traces for wild-type (left) and *PRIP-1 *^*-/- *^(right) showing the shift in baseline holding current elicited by 20 μM BMI and/or 2 μM strychnine (stry). The bar graph shows baseline shift elicited by 20 μM BMI (wild-type, n = 23; *PRIP-1 *^*-/- *^, n = 14) and/or 2 μM strychnine (wild-type, n = 6; *PRIP-1 *^*-/- *^, n = 6). BMI and strychnine were applied for 3-5 min in each experiment. (B) Representative traces and mean sIPSCs for wild-type (left) and *PRIP-1 *^*-/- *^(right) mice showing the sIPSCs elicited by 1 μM DZP. Tau is the decay time constant for the averaged trace of all sIPSCs recorded over 1 min in the absence or presence of DZP. DZP was applied for 8-10 min in each experiment. The red line in the averaged sIPSC trace shows the curve derived from a double-exponential equation. The bar graph shows summary of the tau data. Bars represent means and S.E.M. (*P < 0.05 vs. control, n = 6; †*P < 0.05 *vs. wild-type, n = 6).

Spontaneous inhibitory postsynaptic currents (sIPSCs) were recorded as outward currents from 56 substantia geratinosa (SG) neurons, the lamina II of the spinal dorsal horn, with cells held at 0 mV. Exposure to 20 μM bicuculline methiodide (BMI) shifted the baseline holding current (Figure 3A), and this shift tended to be smaller in *PRIP-1 *^*-/- *^mice (3.7 ± 0.6 pA, n = 14) than in wild-type mice (4.7 ± 1.3 pA, n = 23), although it was not statistically significant. After administering 2 μM strychnine to block glycine receptors, the baseline shift was not significantly different between wild-type (0.8 ± 0.7 pA, n = 4) and *PRIP-1 *^*-/- *^mice (-0.4 ± 1.3 pA, n = 5).

Next, we examined the effects of diazepam (DZP) on GABAergic sIPSCs in SG neurons (Figure 3B). The mean sIPSCs were best fit into a two-exponential equation. Tau2 was significantly increased by 1 μM DZP in wild-type mice (control: 39.3 ± 8.7 ms, n = 6; DZP: 98.4 ± 20.5 ms, n = 6), but not in *PRIP-1 *^*-/- *^mice (control: 51.6 ± 14.6 ms, n = 6; DZP: 44.2 ± 14.7 ms, n = 6). Tau1 was not affected by DZP in wild-type (control: 7.6 ± 1.3 ms, n = 6; DZP: 11.9 ± 2.9 ms, n = 6) or in *PRIP-1 *^*-/- *^mice (control: 12.0 ± 2.1 ms, n = 6; DZP: 10.0 ± 1.9 ms, n = 6). The 10-90% rise time was not significantly affected by DZP in wild-type (control: 2.9 ± 0.6 ms, n = 6; DZP: 2.7 ± 0.6 ms, n = 6) and *PRIP-1 *^*-/- *^mice (control: 2.8 ± 0.4 ms, n = 6; DZP: 2.9 ± 0.5 ms, n = 6). Peak amplitude was also unaffected by DZP in wild-type (control: 17.1 ± 3.3 pA, n = 6; DZP: 11.4 ± 1.2 pA, n = 6) and *PRIP-1 *^*-/- *^mice (control 19.4 ± 5.1 pA, n = 6; DZP: 22.9 ± 6.3 pA, n = 6). Furthermore, frequency was not significantly affected by DZP in wild-type (control: 0.11 ± 0.02 Hz, n = 6; DZP: 0.09 ± 0.02 Hz, n = 6) and *PRIP-1 *^*-/- *^mice (control: 0.3 ± 0.1 Hz, n = 6; DZP: 0.2 ± 0.1 Hz, n = 6).

PRIP was reported to facilitate the transport of the γ2 subunit-containing GABA_A _receptor [25,26]. It is thought that the functions of the GABA_A _receptor are highly influenced by PRIP. GABA plays an important role in nociceptive transmission in the spinal cord as an inhibitory neurotransmitter [13-20]. Thus, we investigated the role of PRIP-1 on acute or chronic pain using *PRIP-1 *^*-/- *^mice. We found that the second phase, but not the first phase, of the licking and biting responses following intraplantar formalin injection was significantly increased in *PRIP-1 *^*-/- *^mice. Intraplantar injection of formalin evokes nociceptive behaviors in rodents such as licking, biting and lifting of the injected paw in a biphasic manner. The first phase of the response is caused by persistent activation and acute sensitization of nociceptors, while the second phase is caused by continual activation of the nociceptors and sensitization of the central synapses via mechanisms triggered by repetitive stimulation during the first phase [28]. Therefore, the hyperalgesic response in *PRIP-1 *^*-/- *^mice induced by intraplantar injection of formalin may occur via central pathways. On the other hand, the thermal sensitivity during the hotplate test at five different temperatures was not significantly different between *PRIP-1 *^*-/- *^mice and wild-type mice. The responses to the hotplate test involve more complex behavior [29,30]. It has been reported that dysfunction of the GABAA receptor can affect heat nociception [31]. However, we found that deficiency in PRIP-1 did not affect responses to noxious heat stimulation. In our *in situ *hybridization experiment, the mRNA expression of γ2 subunit was decreased in the dorsal horn of the spinal cord in *PRIP-1 *^*-/- *^mice, whereas that of β2 subunit was increased. Our results raise the possibility that the activity of the GABA_A _receptor containing the γ2 subunit during thermal nociception in *PRIP-1 *^*-/- *^mice may be compensated for by the GABA_A _receptor composed of the others subunits containing the β2 subunit. Furthermore, although the mechanical paw withdrawal threshold was decreased in *PRIP-1 *^*-/- *^mice, the ipsilateral hypersensitive reaction was more pronounced in *PRIP-1 *^*-/- *^mice after PSNL. These results suggest that tactile allodynia is affected by PRIP-1 deficiency.

The GABA_A _receptor is a heterooligomeric complex of five subunits and the majority of the receptors in the CNS are thought to consist of α1, β2 and γ2 subunits [27]. We found no differences in α1 subunit mRNA expression between *PRIP-1 *^*-/- *^mice and wild-type mice, whereas β2 subunit mRNA expression was significantly greater in *PRIP-1 *^*-/- *^mice than in wild-type mice throughout the spinal cord. Further studies are required to elucidate the mechanism involved in this change in expression. Interestingly, γ2 subunit mRNA expression in the dorsal and ventral horns of the spinal cord was significantly lower in *PRIP-1 *^*-/- *^mice than in wild-type mice. The γ2 subunit is an important factor that regulates the benzodiazepine sensitivity to GABA_A _receptors, as well as receptor expression and trafficking [32]. Thus, it is thought that the assembly of GABA_A _receptors containing the γ2 subunit, as well as the functions of this receptor, are impaired in the spinal cord of *PRIP-1 *^*-/- *^mice. Ataka et al. [33] reported that phasic inhibition is mediated by both GABAergic and glycinergic inhibitory postsynaptic currents, and that the tonic inhibitory currents are mediated by GABA_A _receptors in the dorsal horn lamina II region of the spinal cord. We thus investigated the tonic inhibitory currents in SG neurons of *PRIP-1 *^*-/- *^mice. These currents were achieved by the application of BMI, but not by strychnine, and tended to be smaller in *PRIP-1 *^*-/- *^mice than wild-type mice, although not statistically significant. This suggests that the function of GABA_A _receptors containing the γ2 subunit may be compensated for by the GABA_A _receptor containing β2 subunit in terms of tonic inhibition in *PRIP-1 *^*-/- *^mice. Recently, it is reported that the GABA_A _receptor δ subunit in the spinal cord mediated tonic inhibition and acute nociception [34]. Tonic inhibitory currents mediated by GABA_A _receptors in spinal neurons play an important role in the regulation of acute nociception and central sensitization. Therefore, it is possible that PRIP-1 knockout may partially influence of tonic inhibition with some of GABA_A _receptor subunits. On the other hand, the prolonged Tau2 induced by DZP in wild-type mice was absent in *PRIP-1 *^*-/- *^mice. In previous studies, three types of IPSCs, including glycine receptor-mediated, GABA_A _receptor-mediated and mixed type, were recorded from lamina II neurons [33,35]. GABA_A _receptor-mediated IPSCs showed slow decay time constant, while glycine receptor-mediated IPSCs were rapid. Our results suggest that the assembly of GABA_A _receptor containing γ2 subunit impaired by PRIP-1 knockout may occur the impairment of the function of GABA_A _receptor in the spinal cord. However, tonic inhibition and kinetics of IPSC were not significantly difference between wild-type and *PRIP-1 *^*-/- *^mice. Therefore, it is thought that the hyperalgesic reactions observed in *PRIP-1 *^*-/- *^mice may involve in other factors in addition to the impairment of GABAergic transmission in the spinal cord of these mice.

In conclusion, we have demonstrated that *PRIP-1 *^*-/- *^mice show behavioral abnormalities in response to various noxious stimuli. It is possible that these behaviors in *PRIP-1 *^*-/- *^mice are dependent on changes in the subunits expression and the function of GABA_A _receptor in the spinal cord. Therefore, defects in PRIP-1, which plays a role in the assembly of the GABA_A _receptor, are responsible for abnormal behaviors in response to pain.

## Methods and Materials

### Animals

*PRIP-1 *^*-/- *^mice were used in our experiments. Mice were housed at 24 ± 2°C with a 12/12-h light/dark cycle (lights on at 8:00 am), and were given free access to commercial food and tap water. The experimental procedures were based on the Guidelines of the Committee for Animal Care and Use of Hirosaki University, Hiroshima University and Kyushu University.

### Formalin test

Each mouse was placed in a clear plastic cage at least 30 min before the formalin injection to allow it to adapt to the new environment. A solution of 5% formalin in saline (50 μl) was injected into the plantar surface of the right hindpaw. The number of flinches (rapid paw shaking) of the injected paw was recorded as a pain-related behavior. The total number of hind paw flinches was determined during twelve 5-min intervals for 60 min after formalin injection.

### Hotplate test

The hotplate tests were performed at five different temperatures (46, 48, 50, 52 and 55°C) (Hot Plate Analgesia Meter MK-350C, Muromachi Kikai Co., Ltd, Tokyo, Japan). The latency to licking of the hindpaw was recorded. To prevent tissue damage, the cut-off time was 60 s for 50°C, 40 s for 52°C, and 30 s for 55°C. Animals not responding within the cutoff time were removed and assigned a score of the cutoff time.

### von Frey test

Mechanical sensitivity was determined using von Frey filaments (Semmes-Weinstein monofilaments, Stoelting, IL, USA) with calibrated bending forces (g), as previously described [36]. In brief, the mice were placed individually in a glass cage with a wire-mesh bottom. After the mice had adapted to the testing environment for 60 min, a series of von Frey filaments (0.008, 0.02, 0.04, 0.07, 0.166, 0.407, 0.692, 1.202, 1.479 and 2.0 g) were pressed perpendicularly against the mid-planter surface of the hind paw from below the mesh floor and held for 3-5 s with the filament slightly buckled. Lifting of the paw was recorded as a positive response. The paw withdrawal threshold was defined as the minimum pressure required to elicit a withdrawal reflex of the paw, at least once in five trials.

### PSNL

The mice were anesthetized with an intraperitoneal injection of pentobarbital (60 mg/kg). Under a light microscope (SD30; Olympus, Tokyo, Japan), a tight ligature was made using an 8-0 silk suture around approximately 1/3-1/2 of the diameter of the sciatic nerve located on the right-hand side. This method is similar to that described by Seltzer et al. [37]. In sham-operated mice, the nerve was exposed without ligation.

### *In situ *hybridization

Mice were killed by decapitation. The lower lumbar segment (L3-L5) of the spinal cord was immediately resected and frozen in liquid nitrogen and stored at -80°C. Sections (20 μm thick) were cut from each nerve tissue block through L4 using a microtome cryostat (Microm HM500 OM, Walldorf, Germany). The sections were thaw-mounted onto APS-coated slides (Matsunami, Japan) and stored at -30°C until used for *in situ *hybridization. For *in situ *hybridization histochemistry, specific oligonucleotides were selected from the following sequences: GABA_A _receptor α1 subunit GGGGTCACCCCTGGCTAAGTTAGGGGTATAGCTGGTTGCTGTAGG; GABA_A _receptor β2 subunit TCGTTCCAGGGCGTTGCGGCCAAAACTATGCCTAGGCAACCCAGC; GABA_A _receptor γ2 _[NDS1]_ subunit CATTTGGATCGTTGCTGATCTGGGACGGATATCAATGGTAGGGGC. The oligonucleotide probes were labeled with [^33^P]-dATP (2000 Ci/mmol; {DuPont-NEN}^_[NDS2]_^) and terminal deoxynucleotidyltransferase (Boehringer Mannheim, Germany). The labeled probes were purified using a QIAquick Nucleotide Removal Kit (Qiagen GmbH, Hilden, Germany). Hybridization was performed as previously described [38], except that the purified probes were diluted to a final concentration of 10^7 ^cpm/ml before use. The hybridized sections were exposed to Kodak BioMAX MR films at -80°C and a Kodak MS screen was used to increase the intensity of the hybridization signals. The films were exposed for 7-10 days and then developed. The optical density of the hybridization signals from three sections (the dorsal horn, the intermediate gray matter and the ventral horn of the L4 region) was acquired using an Epson GT-9800 scanner (Epson, Japan). The density was determined using NIH Image (Bethesda, MD, USA) on a Macintosh G5 computer (Apple Computer, Vancouver, WA, USA). The intensity was calculated relative to the background signal.

### Electrophysiology

Transverse spinal cord slices were obtained from the L4 and L5 spinal cords of 5-8-week-old mice. Each mouse was deeply anesthetized with halothane, decapitated, and the lumbar spinal cord (L1-L6) was removed. After removing the pia-arachnoid membrane, 450-μm-thick spinal cord slices were cut on a brain slicer (VIBRATOME _[NDS3]_ 3000 SERIES) in ice-cold oxygenated sucrose-substituted artificial cerebrospinal fluid (ACSF). This solution contained (in mM): 250 sucrose, 2.5 KCl, 1 NaH_2_PO_4_, 1 MgCl_2_, 2.5 CaCl_2_, 25 NaHCO_3 _and 10 glucose. The solution was bubbled with 95% O_2 _and 5% CO_2_. Slices were incubated for at least 1 h in a storage chamber containing ACSF composed of (in mM): 117 NaCl, 3.6 KCl, 1.2 NaH_2_PO_4_, 1.2 MgCl_2_, 2.5 CaCl_2_, 25 NaHCO_3 _and 11 glucose, with 95% O_2_/5% CO_2 _at room temperature before recording. A spinal cord slice was transferred to a recording chamber and perfused (5 ml/min) with ACSF at room temperature. SG neurons were viewed on a monitor via a 40 × water-immersion objective lens with an infrared differential interference contrast filter and a CCD camera (ORCA-ER C4742-95, Hamamatsu Photonics, Shizuoka, Japan). Patch pipettes (resistance: 3-5 MΩ), made from borosilicate glass (1.5 mm O.D, TW150-3, World Precision Instruments, {Inc, USA}_[NDS4]_) was filled with an internal solution containing (in mM): 150 CsCH_3_SO_3_, 5 KCl, 3 MgCl_2_, 0.1 EGTA and 10 HEPES, 3 Mg(ATP)_2 _and 0.4 NaGTP (pH 7.3 with 1 M CsOH). Membrane currents were recorded with cells held at 0 mV using a Multiclamp 700B (Molecular Devices, Sunnyvale, CA, USA) and digitized at 5-10 kHz with DigiData1322A (Molecular Devices). Data were stored and analyzed using pClamp9.2 software (Molecular Devices), Igor Pro software (WaveMetrics, Inc, Lake Oswego, OR, USA) and {NeuroMatic version 2.00 software}_[NDS5]_. Series resistance compensation was not applied.

### Statistical analysis

Statistical analysis of data was performed using the Mann-Whitney *U *-test or Student's *t *-test for comparisons between two groups, or analysis of variance followed by Tukey's test for multiple comparisons. Results are expressed as means and standard error of the mean (S.E.M.). The level of significance was set at *P < 0.05 *.

## Competing interests

The authors declare that they have no competing interests.

## Authors' contributions

KM performed the von Frey test, patch clamp experiments, data analysis, and wrote the manuscript. MT performed *in situ *hybridization experiments. KM, JY and MF participated in data analysis. TK and MH provided *PRIP-1 *^*-/- *^mice and directed the experiments. KM and SU designed and directed the experiments. All authors read and approved the final manuscript.
